# Identification of non-essential loci within the *Meleagrid herpesvirus 1* genome

**DOI:** 10.1186/s12985-015-0362-9

**Published:** 2015-08-27

**Authors:** Robyn N. Hall, Joanne Meers, Elizabeth V. Fowler, Timothy J. Mahony

**Affiliations:** School of Veterinary Science, The University of Queensland, Gatton, QLD 4343 Australia; Poultry CRC, University of New England, Armidale, NSW 2351 Australia; Present address: Commonwealth Scientific and Industrial Research Organisation – Biosecurity Flagship, Black Mountain, 2601 Australia; Animal Science, Department of Agriculture, Fisheries and Forestry, Brisbane, QLD 4072 Australia; Queensland Alliance for Agriculture and Food Innovation, Centre for Animal Science, The University of Queensland, Brisbane, QLD 4072 Australia

## Abstract

**Background:**

*Meleagrid herpesvirus 1* (MeHV-1) infectious bacterial artificial chromosomes (iBACs) are ideal vectors for the development of recombinant vaccines for the poultry industry. However, the full potential of iBACS as vectors can only be realised after thorough genetic characterisation, including identification of those genetic locations that are non-essential for virus replication. Generally, transposition has proven to be a highly effective strategy for rapid and efficient mutagenesis of iBAC clones. The current study describes the characterisation of 34 MeHV-1 mutants containing transposon insertions within the pMeHV1-C18 iBAC genome.

**Methods:**

Tn5 and MuA transposition methods were used to generate a library of 76 MeHV-1 insertion mutants. The capacity of each mutant to facilitate the recovery of infectious MeHV-1 was determined by the transfection of clone DNA into chicken embryo fibroblasts.

**Results:**

Attempts to recover infectious virus from the modified clones identified 14 genetic locations that were essential for MeHV-1 replication in cell culture. Infectious MeHV-1 was recovered from the remaining 14 intragenic insertion mutants and six intergenic insertion mutants, suggesting that the respective insertion locations are non-essential for MeHV-1 replication in cell culture.

**Conclusions:**

The essential and non-essential designations for those MeHV-1 genes characterised in this study were generally in agreement with previous reports for other herpesviruses homologues. However, the requirement for the mardivirus-specific genes *LORF4A* and *LORF5* are reported for the first time. These findings will help direct future work on the development of recombinant poultry vaccines using MeHV-1 as a vector by identifying potential transgene insertion sites within the viral genome.

**Electronic supplementary material:**

The online version of this article (doi10.1186/s12985-015-0362-9) contains supplementary material, which is available to authorized users.

## Background

*Meleagrid herpesvirus 1* (MeHV-1), commonly known as turkey herpesvirus (HVT), is a non-pathogenic avian herpesvirus originally isolated from turkeys in 1969 [1, 2]. The virus is assigned to the genus *Mardivirus*, which also includes oncogenic *Gallid herpesvirus 2* (GaHV-2), the causative agent of Marek’s disease (MD), and the non-oncogenic *Gallid herpesvirus 3* (GaHV-3). Marek’s disease is a highly contagious neoplastic poultry disease of major economic significance worldwide. The close antigenic relationship amongst the mardiviruses has been exploited since the 1970s through the use of MeHV-1 as a live vaccine to reduce production losses resulting from MD [3]. However, despite widespread vaccination with either MeHV-1, bivalent vaccines containing MeHV-1/GaHV-3 or attenuated GaHV-2 strains, MD outbreaks continue to occur. The isolation of GaHV-2 field strains of increased virulence has been correlated with the loss of protective capacity of these vaccines, which reinforces the need for development of improved MD vaccines [4, 5]. It is likely that novel vaccines targeting GaHV-2 will be constructed using recombinant DNA technologies, for which MeHV-1 is ideally suited as a vector candidate.

In addition to its use as a vaccine against MD, MeHV-1 is also widely utilised as a recombinant vaccine vector for poultry diseases such as infectious laryngotracheitis, Newcastle disease, infectious bursal disease and highly pathogenic avian influenza [6–9]. Currently, only a limited number of transgene insertion sites are used in the development of recombinant MeHV-1 based vaccines. Use of a suboptimal insertion site can have a pronounced effect on vaccine efficacy. For example, Gao *et al.* [10] reported a reduced post-challenge mortality with highly pathogenic avian influenza virus when the haemagglutinin gene was expressed from the MeHV-1 *US2* locus compared to the *US10* locus, likely because *in vivo* virus replication was more affected with the disruption of *US10* compared to *US2*. Thus the identification of alternative transgene insertion sites will be useful for the optimisation of MeHV-1 as a vaccine vector.

The MeHV-1 genome is 159,160 bp in length and has a type 4 herpesvirus genomic structure [11, 12]. It comprises a unique long (U_L_) and a unique short (U_S_) region, flanked by terminal/internal repeat long (TR_L_/IR_L_) and short segments (TR_S_/IR_S_), respectively [11, 12]. In addition, the genomic termini comprise telomeric repeats, or a-like sequences, of variable length, which are cis-acting elements involved in genome packaging [13]. These a-like sequences are also present at the IR_L_/IR_S_ junction [11]. For comparative purposes, this article refers to individual genes by their putative human herpesvirus 1 (HHV-1) homologue, using U_L_ and U_S_ nomenclature [14]. Genes unique to mardiviruses are identified by their protein designation, as described in the MeHV-1 reference sequence [Genbank: NC_002641.1] [11]. The complete MeHV-1 genome encodes 79 putative genes [12]. Of these, 73 are single copy; 66 within the U_L_ region and seven in the U_S_ region. The genes *vNR13* and *icp4* are duplicated, with one copy of each located in the IR_S_ and TR_S_ elements. The *US8* gene, encoding the envelope glycoprotein E (gE), spans the TR_S_-U_S_ boundary region; consequently, the gene US8*, located within the IR_S,_ is a truncated duplication of *US8*. Current knowledge of MeHV-1 gene function has largely been extrapolated from studies on GaHV-2, and more broadly from genetic studies of HHV-1 and other herpesviruses. While MeHV-1 is currently utilised as a vaccine vector, a more detailed understanding of the genetic background of this virus is required to facilitate its further development as a vector.

The establishment of infectious bacterial artificial chromosome (iBAC) technologies for herpesvirus mutagenesis has simplified the process of generating modified viruses for functional studies and recombinant vaccine construction. For global genome mutagenesis studies, transposition has previously been proven to be a valuable tool, since the random insertion of transposon sequences allows for the efficient generation of a library of unique insertional mutants. These mutants can then be screened to determine if the transposon insertion affects the replication capacity of the virus in cell culture [15–19]. In this way, non-essential genetic loci can be readily identified, and concurrently tested for their potential to carry transgenes for the subsequent generation of recombinant vaccines.

The aim of this study was to characterise a MeHV-1 iBAC transposition library by determining the site of transposon insertion and the impact on viral replication in cell culture. Overall, twenty non-essential loci were identified within the MeHV-1 genome. Additionally, the requirement for the mardivirus-specific genes *LORF4A* and *LORF5* are reported for the first time.

## Results

### Transposition into a MeHV-1 BAC

The MeHV-1 iBAC clone used in this study was pMeHV1-C18. It has recently been reported that pMeHV1-C18 has an *in vitro* replication capacity similar to wild-type MeHV-1 despite lacking functional copies of seven genes [20]. The construction of a combined Tn5 and MuA transposition library of pMeHV1-C18 has been described previously [21]. Two transposition systems, Tn5 and MuA, were utilised during the generation of this library due to the early finding that the MeHV-1 genome is partially resistant to Tn5 transposition. The MuA transposon construct was engineered to contain an eGFP marker gene. Due to the observed resistance to Tn5 transposition, only a minimal Tn5 transposon construct was successfully utilised for Tn5 transposition. Briefly, the final library contained 76 mutants with insertions mapping to the MeHV-1 genome, disrupting 30 intragenic and six intergenic locations (Table 1; Additional file 1: Supplemental Table S1). Constructs were screened by restriction enzyme analysis and by bi-directional Sanger sequencing outwards from the 5’ and 3’ termini of the transposon insertion [21]. Results from these screening analyses were consistent with a single insertion event occurring within each construct.

**Table 1:** Summary of transposon-mediated gene interruptions within the coding regions of the MeHV-1 infectious clone pMeHV1-C18. The genes affected and encoded gene products are shown

Gene/element	Gene product/function	pMeHV1-C18	GaHV-2^1^	HHV-1^2^	HHV-3^3^	SuHV-1^4^	BoHV-1^5^
*vLip*	Viral lipase	NE	NE	NA	NA	NA	NA
*LORF2*	Unknown	NE	E/NE	NA	NA	NA	NA
*UL6*	Portal protein	E	ND	E	E	ND	E
*UL8*	Helicase/ primase associated protein	E	ND	E	E	ND	E
*UL9*	Origin binding protein	E	ND	E	E	E	E
*UL10*	Glycoprotein M	NE	E	NE	E	E	NE
*UL13*	Protein kinase	A	NE	NE	NE	NE	NE
*UL17*	Tegument/DNA packaging protein	E	E	E	E	E	E
*UL19*	VP5 capsid protein	E	E	E	E	NE	E
*UL21*	Tegument protein	A	ND	NE	E	NE	NE
*UL26*	Scaffold protease	E	ND	E	E	ND	E
*UL26.5*	Scaffold protein	E	ND	E	E	ND	E
*UL27*	Glycoprotein B	E*	E	E	E	ND	E
*UL29*	Single stranded DNA binding protein	E	ND	E	E	E	E
*UL36*	Large tegument protein	E	E	E	E	E	E
*UL37*	Tegument protein	E	E	E	E	ND	E
*UL39*	Ribonucleotide reductase large subunit	NE	NE	NE	NE	NE	NE
*UL40*	Ribonucleotide reductase small subunit	NE*	ND	NE	NE	ND	NE
*UL42*	DNA polymerase processivity subfactor	E	ND	E	E	ND	E
*UL47*	VP13/14 capsid protein	NE	NE	NE	NE	NE	NE
*UL48*	VP16 α-transinducing factor	NE	NE	E	NE	NE	NE
*UL52*	Helicase/ primase associated protein	E	ND	E	E	ND	E
*UL53*	Glycoprotein K	A	E	NE	E	E	E
*LORF4A*	Unknown	NE	ND	NA	NA	NA	NA
*LORF5*	Unknown	NE#	NE	NA	NA	NA	NA
*icp4*	Major immediate early regulatory gene	E	ND	E	E	ND	ND
*US3*	Protein kinase	NE	NE	NE	E	NE	NE
*US6*	Glycoprotein D	NE*	NE	E	ND	NE	E

The gene requirements for pMeHV1-C18 determined in this study are designated as either essential (E), non-essential (NE) or severely attenuated (A). Comparative data, using the same nomenclature, is shown for selected alphaherpesviruses; *Gallid herpesvirus 2* (GaHV-2), *Human herpesvirus 1* (HHV-1), *Human herpesvirus 3* (HHV-3), *Suid herpesvirus 1 (*SuHV-1) and *Bovine herpesvirus 1 (*BoHV-1). Not applicable (NA) denotes genes unique to the genus *Mardivirus*. Rows in bold highlight those genes for which requirement varies between the viruses listed. *Requirement for growth in cell culture previously reported in wildtype MeHV-1 virus [57–59]

#Transposon was unstable in viral genome

References for gene interruption studies: ^1^[18, 23, 25, 42, 49, 57, 60–67]; ^2^[14, 35, 38, 58, 68–76]; ^3^[26, 41, 45, 77, 78]; ^4^[17, 27, 43, 48, 79–82]; ^5^[19, 83]

**Fig. 1:**
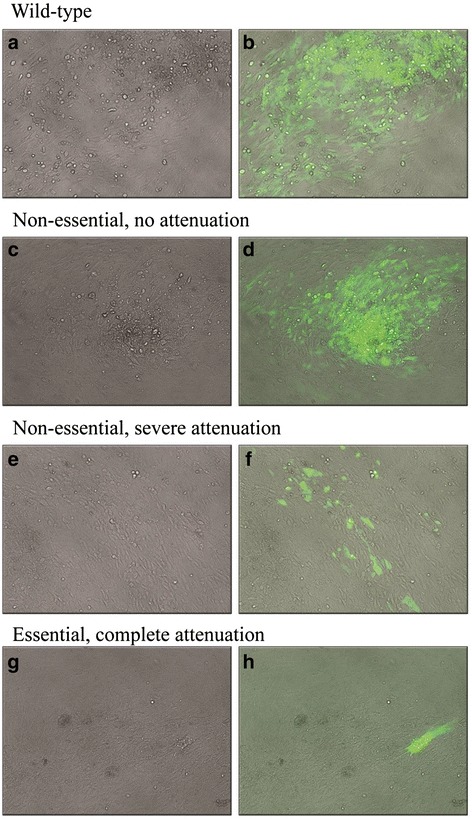
Classification of the replication capacity of transposition mutants of MeHV-1 in cell culture. Gene requirements of MeHV-1 were assigned based on the replication capacity of the respective transposition mutants in cell culture when compared to parental virus (MuAΔ65 day 5 post-transfection **a**: brightfield × 100; **a**nd **b**: fluorescent microscopy × 100). Clones were classified as ‘non-essential, no attenuation’ (MuAΔ72 day 5 post-transfection **c**: brightfield × 100; and **d**: fluorescent microscopy × 100), ‘non-essential, severe attenuation’ (MuAΔ68 day 7 post-transfection **e**: brightfield × 100; and **f**: fluorescent microscopy × 100), or ‘essential, complete attenuation’ (MuAΔ64 day 7 post-transfection **g**: brightfield × 100; and **h**: fluorescent microscopy × 100). BAC DNA encoding the MeHV-1 genome and containing a single transposon insertion within either the BAC vector backbone, therefore reflecting parental virus (**a** and **b**), *UL48* (**c** and **d**), *UL53* (**e** and **f**) or *UL27* (**g** and **h**) was transfected into CEFs. Monolayers were passaged every five to eight days and were observed for the development of CPE using light microscopy (**a**, **c**, **e** and **g**) and, whenever possible, for expression of a marker gene (eGFP) using fluorescent microscopy (**b**, **d**, **f** and **h**)

Forty-seven insertion events mapped to within the U_L_ genomic region, three events mapped to the U_S_ region, two insertions were identified within the IR_L_/TR_L_ regions and 22 insertions mapped to the IR_S_/TR_S_ regions. Additionally, two insertions mapped to the a-like sequences. Recovery of virus from transposed clones was assessed by observing characteristic MeHV-1 cytopathic effect (CPE) using light microscopy, and whenever possible, by detecting the expression of enhanced green fluorescent protein (eGFP) using fluorescent microscopy (the MuA transposon used to generate MuAΔ48-84 contained an eGFP transgene) (Fig. 1). Cell monolayers were passaged at least once after CPE was evident to confirm the presence of infectious virus. Where CPE was not observed, monolayers were blind passaged three to four times to confirm the absence of infectious virus.

### Transposition into the U_L_ region

Within the U_L_ genomic region, 25 genes were disrupted by transposon insertion events. Of these, 13 and 12 locations were found to be essential and non-essential for replication of MeHV-1 in cell culture respectively (Table 1).

Although *UL13* and *UL53* transposition mutants were classed as non-essential based on the observation of persistent eGFP expression after multiple passages, the recovered viruses from these clones showed severely attenuated replication in cell culture compared to the parental virus (Fig.1e and 1f, Table 1).

The *UL21* disruption mutants, Tn5Δ14, MuAΔ37 and MuAΔ41, also caused a very low grade CPE compared to the parental virus. As these constructs were generated with transposons lacking the eGFP reporter gene, expression of eGFP could not be used to verify virus replication in these clones. Instead, these clones were confirmed to facilitate the recovery of infectious MeHV-1 through detection of viral DNA in the cell monolayer using PCR after three or more passages. To exclude the possibility that the PCR assay was amplifying residual transfected iBAC DNA, the pMeHV1-C18-MuAΔ64 construct, a non-infectious glycoprotein B disruption mutant (Fig. 1g and 1h), was assayed in parallel with the same PCR assay. As expected, no pMeHV1-C18-MuAΔ64 DNA was detectable at passage three, confirming that DNA detected from *UL21* disruption mutants was due to ongoing viral replication. Based on the continued detection of viral DNA, the *UL21* insertion site was designated as non-essential for MeHV-1 replication in cell culture, although replication was markedly impaired.

The *LORF5* gene was found to be non-essential for viral replication in cell culture, since CPE was observed for the disruption mutants pMeHV1-C18-MuAΔ59 and MuAΔ82. However, both mutants were observed to partially lose eGFP expression by passage three in cell culture, based on the presence of both eGFP expressing foci of CPE as expected, as well as non-eGFP expressing foci.

### Transposition into the U_S_ region

Two genes within the U_S_ region, *US3* and *US6*, were disrupted by transposon insertion events. Both insertion locations were determined to be non-essential for MeHV-1 replication in cell culture (Table 1).

### Transposition into the IR_S_/TR_S_

As previously reported, the IR_S_/TR_S_ of MeHV-1 were transposed at higher frequency compared to the rest of the viral genome [21]. Of the 76 pMeHV1-C18 transposition mutants generated, 22 (29 %) mapped to the IR_S_/TR_S_ regions of the genome. Eleven of these insertions were within the *icp4* coding region. Three clones, pMeHV1-C18-Tn5Δ13, Tn5Δ21 and MuAΔ76, mapped within the repeated segment of the *US8* gene. The transposition events in seven clones were mapped to non-coding sequences of the IR_S_/TR_S_ elements. The remaining clone, pMeHV1-C18-MuAΔ46, contained a transposon insertion within the dual-copy gene *vNR13*.

**Fig. 2:**
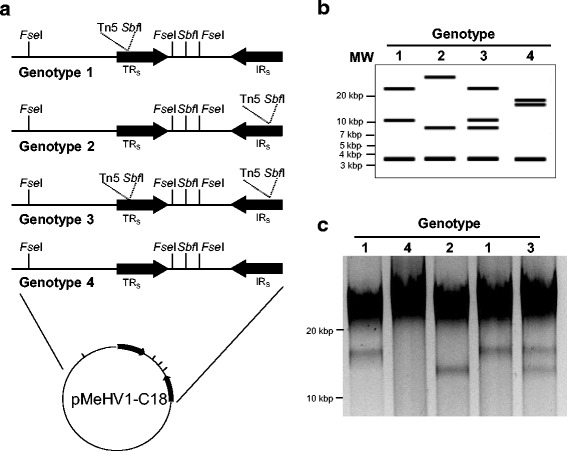
Restriction fragment length polymorphism of *icp4* insertion mutants **a** Schematic representation of the four potential genotypes resulting from recombination between the IR_S_/TR_S_ elements of pMeHV1-C18-Tn5Δ-1 during virus replication. Genotype 1: Transposon in TR_S_; Genotype 2: Inversion of transposon to the IR_S_; Genotype 3: Duplication of transposon; Genotype 4: Loss of transposon. (**b**) *In silico Fse*I/*Sbf*I restriction endonuclease digestion patterns for pMeHV1-C18-Tn5Δ1 Genotypes 1, 2, 3, and 4. (**c**) iBAC DNA co-digested with *Fse*I and *Sbf*I. lane 1: pMeHV1-C18-Tn5Δ1 (genotype 1); lane 2: loss of transposon (genotype 4); lane 3: transposon in IR_S_ element (genotype 2); lane 4: transposon in TR_S_ element (genotype 1); lane 5: duplication of transposon (genotype 3)

All insertions mapped to different nucleotide positions within the IR_S_/TR_S_ elements, demonstrating that these clones originated from independent transposition events (Additional file 1: Supplemental Table S1). Insertion events generated using the Tn5 < KAN-2 > transposon were mapped to a specific repeat based on the presence of suitable restriction endonuclease (RE) cleavage sites within both the transposon construct and the iBAC (Fig. 2). Interestingly, of the 10 insertions generated by Tn5 transposition that mapped to the IR_S_/TR_S_ regions, eight were within the TR_S._ Virus was successfully recovered from all Tn5 transposon mutants with insertions in the IR_S_/TR_S_. However, this did not reflect the requirement of the genetic elements at the location of the insertion for viral replication, since the untransposed copy of the gene may compensate for loss of the transposed copy. Furthermore, it was anticipated that recombination between the IR_S_ and TR_S_ elements during viral replication could result in the recovery of the parental iBAC genotype (Fig. 2).

In an attempt to determine the requirement of dual-copy genes, naturally occurring recombination between the IR_S_/TR_S_ elements during viral replication was exploited to isolate double-insertional mutants [22]. This was investigated using the *icp4* disruption mutant, pMeHV1-C18-Tn5Δ1. It was postulated that following recovery of infectious MeHV-1 and subsequent viral replication, recombination between the IR_S_ and TR_S_ regions during replication would result in the generation of four genotypes as illustrated in Fig.2a. These genotypes were: **genotype 1**, a single transposon insertion in the TR_S_ as described for pMeHV1-C18-Tn5Δ1; **genotype 2**, characterised by an inversion of the repeat sequence elements, transferring the original transposon insertion to the IR_S_ element; **genotype 3**, replacement of the unmodified IR_S_ with the transposed TR_S_ sequence caused a duplication of the TR_S_ transposon element, thus generating a double *icp4* disruption mutant; or **genotype 4**, a duplication of the IR_S_ element eliminating the transposed TR_S_ element, thereby restoring the parental iBAC sequence. The requirement of *icp4* for replication can be assessed using a genotype 3 construct, because of its *icp4*-negative genotype (Fig.2a).

To determine which of these genotypes could be recovered from cells infected with virus recovered from pMeHV1-C18-Tn5Δ1, total DNA was isolated and electroporated into bacterial cells. Putative iBAC DNA were recovered from chloramphenicol resistant bacteria and digested with *Fse*I and *Sbf*I. *In silico* analyses suggested that double-digestion of Tn5 transposition mutants with these restriction enzymes would generate fragment profiles characteristic for the genotypes described previously (Fig.2b). All four genotypes could be distinguished by the presence or absence of two polymorphic fragments with a predicted size of ~12kbp and ~8.5kbp.

The RE patterns of five recovered pMeHV1-C18-Tn5Δ1 iBAC clones digested with *Fse*I and *Sbf*I are shown in Fig.2c. The predicted polymorphic fragments generated by the addition of the *Sbf*I site by Tn5 transposition into the TR_S_/IR_S_ elements were clearly distinguishable, although their estimated sizes were larger than expected (~14.1kbp and ~12.5 kbp; Fig.2c). There are several factors that may have retarded the migration of these fragments including overloading of iBAC DNA, presence of impurities or excess bacterial host DNA. All potential genotypes generated two large fragments, represented by the large fluorescent bands (Fig.2b and 2c), which are not individually resolvable by standard agarose gel electrophoresis. The predicted 3.7-kbp and 3.6-kbp fragments were not observed in the electrophoretic analyses due to their relatively small size; thus these fragments represented only a very small proportion of the total DNA and their staining was expected to be beyond the limits of detection. As these fragments were identical in all potential genotypes and therefore visualisation of these fragments was not necessary for evaluation of the assay.

Recovered clones that had the larger polymorphic fragment were classified as genotype 1, while clones with the smaller polymorphic fragment were identified as genotype 2. Clones with both fragments were designated as *icp4-*negative mutants (genotype 3). Clones with neither of these fragments had reverted to the genotype of the parental iBAC (genotype 4). Of the 41 clones subjected to digestion with *Sbf*I and *Fse*I, 10 clones were classified as genotype 1, 11 clones were genotype 2, five clones were determined to be of genotype 3 and 10 clones were genotype 4. The RE fragment profiles of the remaining five clones analysed were inconsistent with the parental clone, pMeHV1-C18-Tn5Δ1, and were discarded from further analyses.

One dual-*icp4* disruption clone (genotype 3), pMeHV1-C18-Tn5Δ1-3, was used to investigate the requirement for *icp4* for MeHV-1 replication in cell culture. Transfection of this construct into chicken embryo fibroblasts (CEFs) did not result in any observable CPE after three passages, demonstrating that *icp4* is essential for MeHV-1 replication in cell culture. Although the approach described above resulted in the isolation of a dual-copy *icp4* disruption mutant, it was not successfully applied to the isolation of dual-copy mutants for other genes in IR_S_/TR_S_ regions.

### Transposition into the IR_L_/TR_L_

Two MuA transposon insertion events mapped to the non-coding sequences of the IR_L_/TR_L_ regions. Since insertions events were isolated from MuA transposition reactions, they could not be localised to individual repeats due to lack of suitable RE sites in the transposon construct, and dual-copy disruption mutants could not be not generated.

### Transposition into a-like sequences

Two transposed clones, pMeHV1-C18-Tn5Δ16 and Tn5Δ20, contained insertions within the a-like sequences of the MeHV-1 genome (Additional file 1: Supplemental Table S1). The exact nucleotide position of the insertion could not be determined for these clones, since the nucleotide sequence data did not extend beyond the termini of this repetitive sequence element. Transfection of Tn5Δ16, with a single transposon insertion into an a-like sequence, produced CPE indistinguishable from that of wild-type MeHV-1. However, because only one copy of the a-like sequences was disrupted, the requirement of these regions for replication of MeHV-1 in cell culture could not be determined in the current study.

## Discussion

The requirements for 28 coding and six non-coding regions within the MeHV-1 genome have been determined in this study, using a library of 76 transposition mutants. These included 11 genes for which the *in vitro* requirements for replication have not previously been reported in any of the mardiviruses (Table 1). Clones with unique phenotypes and gene designations that contrast with the reported literature are discussed in detail below. Of particular interest in this study were those insertion sites that were non-essential for replication, as these represent potential transgene insertion sites for MeHV-1-based vectors. These sites included 14 intragenic and six intergenic sites.

Homologues of the *LORF2* gene are restricted to the *Mardivirus* genus. It has been suggested that this gene may have a role in mRNA transcription or processing but this is yet to be experimentally confirmed [12]. Recently, the GaHV-2 *LORF2* homologue was reported to have immunoevasion functions via the down-regulation of MHC class I in infected cells [23]. *LORF2* has previously been reported as essential for GaHV-2 replication in cell culture [18]. In contrast, another study reported retroviral insertions within the ORF as having no effect on GaHV-2 replication [23, 24]. In the current study, transposition clone MuAΔ30 contained an insertion within the first exon of MeHV-1 *LORF2*, disrupting 99.5 % of the predicted polypeptide. Viral recovery experiments clearly demonstrated that this location was non-essential for replication of MeHV-1 in cell culture, with CPE developing within five days of transfection. The differing requirement of this gene in MeHV-1 and GaHV-2 is of interest, and further investigations into LORF2 function in MeHV-1 are warranted. Furthermore, the use of *LORF2* as a transgene insertion site for vaccine development may increase vaccine efficacy through impairment of the proposed LORF2 immunoevasion functions.

The non-essential classification of MeHV-1 *UL10* in this study contrasts with the essential assignment of the GaHV-2 *UL10* homologue (Table 1) [25]. The *UL10* gene encodes a homologue of glycoprotein M (gM), a core herpesvirus gene [11, 12]. The *UL10* gene is essential in the strictly cell-associated viruses GaHV-2 and *Human herpesvirus 3* (HHV-3) [25, 26]. In contrast, *UL10* has consistently been reported as non-essential for viral replication in cell culture for cell-free herpesviruses such as HHV-1, *Suid herpesvirus 1* (SuHV-1), *Bovine herpesvirus 1* (BoHV-1), *Equine herpesvirus 1* (EHV-1) and *Gallid herpesvirus 1* [19, 27–30]. Although the parental MeHV-1 strain FC126 used in this study was cell-associated, cell-free virus is produced to a limited extent and this strain can be adapted to produce high titres of cell-free virus [31]. It has also been suggested that expression of glycoprotein D (gD) may compensate for loss of gM function, since both HHV-3 and GaHV-2 do not express gD in cell culture, and this may explain the essential designation of gM in these viruses [25]. While the expression of MeHV-1 gD during infection in cell culture has not been reported to date, the capacity of the virus to adapt to cell-free growth suggests it is.

The *UL21* gene encodes a poorly characterised tegument protein that is capsid-associated and may have roles in intracellular transport and in nuclear egress [32, 33]. The recovery of infectious MeHV-1 from three *UL21* transposed clones, Tn5Δ14, MuAΔ37 and MuAΔ41, in combination with the presence of viral DNA after sequential passages, confirms that MeHV-1 *UL21* is non-essential for replication in cell culture. However, replication was severely attenuated compared to the parent virus. Disruption studies in other alphaherpesviruses have shown *UL21* to be non-essential, although a range of deleterious effects have been noted on virus replication (Table 1). A *UL21* mutant of SuHV-1 showed impaired replication in cell culture and reduced virulence *in vivo* [17, 34]. For HHV-1 and BoHV-1, *UL21* has been shown to be non-essential, but deletion reduced the *in vivo* replication capacity of HHV-1 [19, 35, 36]. In contrast, *UL21* has been reported to be essential for *Human herpesvirus 2*, HHV-3 and EHV-1 [26, 32, 37]. The severe attenuation observed for MeHV-1 in this study suggests the *UL21* gene is unsuitable for use in recombinant vaccine applications, however it may be of use for generating replication-limited gene delivery constructs for poultry research applications.

The non-essential phenotype of MeHV-1 *UL48* disruption mutants characterised in this study conflicts with the essential requirement of this gene for HHV-1 replication (Fig.1c and 1d). In HHV-1, *UL48* encodes the VP16 α-trans inducing factor, a tegument protein that induces immediate-early gene transcription and is also required for virion assembly [38]. The *UL48* homologues of many alphaherpesviruses, including mardiviruses, lack the acidic carboxyl terminus transactivating domain present in the HHV-1 *UL48* protein, however transactivating functions may be retained via other transactivation sites within *UL48* [11, 12, 39, 40]. This gene is essential for the replication of HHV-1 and EHV-1 in cell culture, but is non-essential in other alphaherpesviruses investigated to date, including HHV-3, SuHV-1, BoHV-1 and GaHV-2 (Table 1) [19, 26, 38, 41–45].

The MeHV-1 *UL53* gene is a homologue of the HHV-1 gene encoding glycoprotein K (gK) [11, 12]. Similar to other viral glycoproteins, gK has roles in cell-to-cell fusion and in viral egress from infected cells [46, 47]. It has been reported to be essential for replication of many alphaherpesviruses, including GaHV-2, HHV-3, SuHV-1 and BoHV-1 (Table 1) [19, 26, 48, 49], while it is non-essential for HHV-1 and EHV-1 growth *in vivo* [50, 51]. Interestingly, deletion of *UL53* from both the HHV-1 and EHV-1 genomes resulted in severely attenuated viruses with greatly reduced plaque sizes and impaired virion penetration in cell culture [37, 51, 52]. Marked attenuation was also observed for the MeHV-1 *UL53* disruption mutant, MuAΔ68, in this study (Fig.1e and 1f).

The MeHV-1 *LORF4A* gene is a homologue of *LORF4* genes of GaHV-2 and GaHV-3 and *LORF9* of *Anatid herpesvirus 1*, and the encoded polypeptide shares 47 % amino acid identity to the proposed paralogue, MeHV-1 LORF4 [11, 12]. The *LORF4* homologues have been postulated to have roles as avian host range determinants, since the occurrence of this gene is restricted to mardiviruses [11, 12]. Transposon insertion into *LORF4A* in the pMeHV1-C18 transposon clone Tn5Δ10 disrupted 82 % of the gene, and the insertion location was designated as non-essential for virus growth in cell culture. This is the first report of a disruption mutant of *LORF4*.

Overall fourteen genetic locations were identified as essential for MeHV-1 replication in cell culture (Table 1)*.* The classification of these loci provides additional foundational information concerning MeHV-1 replication, as the requirements of 13 of these genes have not previously been reported for MeHV-1. Although it would have been interesting to determine the effects of insertions on global viral gene expression and protein production, this was beyond the scope of the current study. Similarly, revertant constructs were not generated for replication-defective mutants since putatively essential genes are not of further interest for vaccine development.

It is noteworthy that the transposition mutants reported here are cumulative gene deletion mutants of MeHV-1, as pMeHV1-C18 lacks seven coding regions compared to the parental virus [20]. This genetic background may have contributed to the observed attenuation of some clones compared to the wild-type MeHV-1. It is considered unlikely that the requirement of the non-essential loci identified in this study would be essential in the full-length virus, as it is reasonable to conclude that effects on viral replication are likely to be more severe with cumulative gene deletions compared to the disruption of a single gene. However, it is possible the locations designated as essential in this study may be non-essential in the parental virus. Nonetheless, this is also considered unlikely as the MeHV-1 genes designated as essential in this study conform with the reported requirements for the respective homologues of other alphaherpesviruses, with the exception of *UL19* which is reported as non-essential in SuHV-1 (Table 1) [17]. However, it must be noted that in that study, the transposon insertion event mapped 2 bp downstream of the SuHV-1 *UL19* ORF, therefore it could be argued that this was not a true report of the UL19 requirement in this virus as complete translation of the encoded polypeptide would have been possible.

Given the instability observed in the *LORF5* insertion mutants, it is possible that this is an essential gene and it may have been misclassified as non-essential in the current study. This is considered unlikely, since in the case of the *LORF5* mutants, the transgene was gradually lost during serial passage of recovered virus. In the case of an insertion into an essential gene, the insertion mutant would not undergo sufficient replication capacity to facilitate loss of the transgene and subsequent recovery of virus. Regardless of whether the *LORF5* gene is essential or non-essential for replication, the observed instability of the transposon insertions in two independent *LORF5* transposon insertion mutants suggests this region of the genome would be unsuitable for recombinant vector applications.

Despite the potential limitations of the cumulative gene deletion genotype of the iBAC used in this study, it has enabled the identification of viruses with novel phenotypes, for example the MuAΔ68 virus with an insertion into *UL53*. While CPE was observed, it was subtle compared to the parent virus and may have been missed completely in the absence of reporter gene expression (Fig.1e and 1f). It is considered highly unlikely that a virus with this phenotype could be constructed using rational gene-targeting strategies.

Importantly, potential insertion sites for vector development must also be verified *in vivo,* since it is generally accepted that non-essential genes in cell culture may have major roles *in vivo*, for example in immunoevasion or other virus-host interactions [14]. An example of this are glycoprotein C (gC)-null mutants of GaHV-2, which show increased viral replication in cultured cells*,* however *in vivo* infection required a longer incubation period to establish infection, viraemia and induction of seroconversion compared to gC-positive virus, and gC-null viruses were not transmitted horizontally [53, 54]. The *in vivo* replication capacity of virus recovered from the parental iBAC used in this study is reduced compared to wild-type MeHV-1 [20]. As a result it might be expected that any constructs derived from this parent clone would be further attenuated *in vivo*. Extrapolating from the GaHV-2 studies discussed above, a deletion identified in the *UL44* region of pMeHV1-C18 likely contributes to the *in vivo* attenuation observed with this construct. Therefore consideration should be given to the restoration of this deletion prior to *in vivo* assessment of the non-essential gene mutants constructed in the current study.

The strategy used to determine the replication requirement for *icp4* highlights the power of iBAC technologies, for example to generate a dual-disruption mutant with two mutagenised copies of a repeat element. This strategy was developed after the generation of the transposition libraries, thus the presence of suitable RE sites in the Tn5 transposon and the virus was serendipitous. Future studies investigating genetic elements located in the repeat sequences of herpesvirus iBACs should consider the identification of suitable RE sites within the targeted viral genome to enable the identification of modified specific repeat sequences. If appropriate sites are identified in the virus, complementary sites could be readily incorporated into the proposed transgene molecule to facilitate the isolation of double-deletion/disruption mutants.

## Conclusions

Despite the previously reported resistance of the MeHV-1 genome to transposition [21], characterisation of mutant clones obtained using these methods has enabled determination of twenty non-essential genomic locations. When considered together with the parent genotype of the MeHV-1 iBAC used in this study, these results demonstrate the considerable degree of redundancy of genes within the MeHV-1 genome *in vitro*. Moreover, the genotype of pMeHV1-C18, containing multiple deletions compared to the reference MeHV-1 genome, has enabled the identification of viruses with unique phenotypes, such as the gK and UL21 disruption viruses, which replicated in the virtual absence of CPE. Of interest in future studies would be the sequential restoration of genes into these replication-impaired viruses to determine which genes restore the capacity of recovered virus to cause a CPE more characteristic of MeHV-1.

## Methods

### Transposition libraries

Construction and characterisation of the MeHV-1 iBAC clone, pMeHV1-C18, is described in Mahony *et al.* [20]. This iBAC contains cumulative gene deletions compared to the MeHV-1 FC126 strain, along with BAC vector sequences within the *SORF3/US2* region, and is genetically defined as pMeHV1-C18Δ*UL43:UL44:UL45:UL56:pp38:SORF3:US2,* however the short form (pMeHV1-C18) will be retained in this manuscript for simplicity. The generation of pMeHV-C18 transposition clones characterised in this study was reported previously [21].

### Restriction endonuclease analyses

Double digestion of Tn5-transposition mutants was performed with *Sbf*I and *Fse*I at 37 °C for 1 h, and reactions were heat-inactivated at 65 °C for 20 min. Digestion products were resolved at 60 V for 3 h in 0.7 % agarose gels in 1 × Tris-acetate-EDTA buffer containing 0.1 μg mL^−1^ ethidium bromide. Banding patterns were visualised using UV light.

### Identification of transposition insertion site and orientation

To identify transposon insertion location within the parent iBAC, bi-directional sequencing was performed using primers specific for the respective transposons (Additional file 1: Supplemental Table S2). Data were analysed using 4Peaks software (http://www.mekentosj.com/science/4peaks) and mapped to the MeHV-1 genome using Blastn analyses [55].

### Growth of pMeHV1-C18 transposition mutants in cell culture

To assess the capacity of the transposed clones to facilitate the recovery of infectious MeHV-1, DNA was prepared from transposon clones and transfected into CEFs. The CEF cells were maintained in a 5 % CO_2_ environment at 37 °C in Medium 199 (Gibco), containing 10 % foetal bovine serum (Gibco) and 1 × Antibiotic-Antimycotic (Gibco). Recovered iBAC DNA was transfected in triplicate into CEFs at 80 % confluency, using Lipofectamine and Plus reagent (Invitrogen) according to manufacturer’s instructions. Recovery of virus was assessed on a daily basis for characteristic MeHV-1 CPE using light microscopy, and where possible, by the presence of eGFP expression using fluorescent microscopy. Monolayers were passaged at least once after CPE was evident to confirm the presence of passageable virus. Monolayers in which CPE was not observed, were blind passaged after five to eight days at least three times to confirm the absence of infectious MeHV-1.

### Analyses of *UL21* disruption mutants

PCR for detection of *UL21* insertion clones was performed on total DNA harvested from infected cultures at the third or sixth passage, as described previously [56]. Total DNA was extracted using the DNeasy blood and tissue kit (Qiagen). Elute (2 μL) was used as a template for PCR over the *UL21* coding region using oligonucleotide pairs: HVT27Fwd with HVTFrag5Rev, HVT27Fwd with KanME-Rev, and KanME-Fwd with HVTFrag5Rev (Additional file 1: Supplemental Table S2). Each 50 μL PCR reaction contained 1 × PCR buffer – Mg (Invitrogen), 0.2 mM each dNTP, 1.5 mM MgCl_2_, 0.2 μM each primer, 1 U Platinum *Taq* DNA polymerase (Invitrogen) and 2 μL template. Cycling conditions were: 94 °C for 2 min followed by 30 cycles of 94 °C for 30 s, 55 °C for 30 s, 72 °C for 1 min per kbp. PCR products (5 μL) were resolved on 1 % agarose gels containing GelRed at 60 V for 1 to 1.5 h.

### Characterisation of transposition events in genomic repeat regions

BACs were re-isolated from cultures infected with the Tn5 mutant pMeHV1-C18-Tn5∆1, containing a transposon insertion within the IR_S_ copy of *icp4*. Briefly, total DNA was extracted from cultures four days after the third passage, as described previously [56]. Purified DNA (1 μg) was electroporated into 50 μl of Electromax DH10B *E. coli* (Invitrogen) and selected for on Luria-Bertani (LB) agar plates containing either 12.5 μg mL^−1^ chloramphenicol alone, or with the addition of 30 μg mL^−1^ kanamycin. Antibiotic-resistant colonies containing an iBAC construct were isolated, and DNA was extracted and double-digested with *Fse*I and *Sbf*I as described above. A genomic sequence for pMeHV1-C18-Tn5Δ1 was generated *in silico* by insertion of the Tn5 transposon sequence to simulate the TR_S_ insertion genotype. Analogous genomic sequences were also generated for clones with the IR_S_ insertion and the IR_S_/TR_S_ dual insertion genotypes. Selected clones representing the dual-*icp4* deletion genotype were identified by restriction endonuclease digestion and subsequently transfected back into CEFs, and cultures were monitored for the development of CPE.

## Additional file

Additional file 1:
**Supplemental Table S1.** Details of transposon gene interruptions into the MeHV-1 infectious clone pMeHV1-C18. Supplemental Table S2: Oligonucleotides used in this study. Supplemental Figure S1: Primary chicken embryo fibroblasts (CEFs) were used as a negative control. (PDF 121 kb)

## Abbreviations

BoHV-1: Bovine herpesvirus 1
CEFs: Chicken embryo fibroblasts
CPE: Cytopathic effect
EHV-1: Equine herpesvirus 1
eGFP: Enhanced green fluorescent protein
GaHV-2: Gallid herpesvirus 2
GaHV-3: Gallid herpesvirus 3
gC: Glycoprotein C
gD: Glycoprotein D
gE: Glycoprotein E
gK: Glycoprotein K
gM: Glycoprotein M
HHV-1: Human herpesvirus 1
HHV-3: Human herpesvirus 3
HVT: Turkey herpesvirus
iBAC: Infectious bacterial artificial chromosome
IRL: Internal repeat long
IRS: Internal repeat short
MD: Marek’s disease
MeHV-1: Meleagrid herpesvirus 1
RE: Restriction enzyme
SuHV-1: Suid herpesvirus 1
TRL: Terminal repeat long
TRS: Terminal repeat short
UL: Unique long
US: Unique short
