# Supplementary material for: Identification of non-essential loci within the Meleagrid herpesvirus 1 genome
Source: Virol J. 2015 Aug 27;12:130. doi: 10.1186/s12985-015-0362-9 (PMC4550065; doi:10.1186/s12985-015-0362-9)

Supplemental Table S1: **Details of transposon gene interruptions into the MeHV-1 infectious clone pMeHV1-C18.**

The clone name, gene designation and nucleotide position of the transposon insertion are detailed. Insertion locations were mapped to the MeHV-1 reference genome sequence [Genbank: NC_002641.1]. The orientation of the transposon insertion into the parent iBAC genome, either forward (+) or reverse (-) in respect to the MeHV-1 reference genome sequence, was determined by DNA sequencing. Insertions within repeat regions of Tn5 insertion clones were further characterised by restriction enzyme analysis with *Sbf*I and *Fse*I.

Gene/ element	Clone	Nucleotide position (+/-)
vLip (HVT005)	MuAΔ38	7047-7051 (+)
LORF2 (HVT007)	MuAΔ30	9327-9331 (+)
UL6	MuAΔ75<br>MuAΔ73	17747-17751 (+)<br>17873-17877 (+)
UL8	MuAΔ58	20160-20164 (-)
UL9	Tn5Δ2<br>MuAΔ29	22352-22360 (+)<br>23883-23887 (-)
UL10	MuAΔ28<br>MuAΔ48	25857-25861 (+)<br>26030-26034 (+)
Intergenic	MuAΔ74	26188-26192 (+)
UL13	MuAΔ69	28881-28885 (+)
UL17	MuAΔ27<br>Tn5Δ12<br>MuAΔ32	32633-32637 (+)<br>33504-33512 (-)<br>34287-34291 (-)
UL19	MuAΔ66	40885-40889 (+)
UL21	MuAΔ37<br>Tn5Δ14<br>MuAΔ41	42375-42379 (+)<br>42635-42643 (-)<br>43080-43084 (-)
UL26	MuAΔ53	50761-50765 (-)
UL26.5	MuAΔ31	51870-51874 (-)
UL27	MuAΔ43<br>MuAΔ64<br>MuAΔ33	53097-53101 (-)<br>54786-54790 (+)<br>55039-55043 (-)
UL29	Tn5Δ7<br>MuAΔ26	58098-58106 (+)<br>59940-59944 (-)
Intergenic	MuAΔ40	68440-68444 (+)
UL36	MuAΔ81<br>MuAΔ52<br>MuAΔ79	72199-72203 (+)<br>72288-72292 (-)<br>78147-78151 (-)
UL37	Tn5Δ6	80288-80296 (+)
UL39	Tn5Δ23	86217-86225 (-)
UL40	Tn5Δ18	87217-87225 (+)
Intergenic	MuAΔ35	89528-89532 (-)
UL42	MuAΔ42	90563-90567 (+)
UL47	MuAΔ80	98798-98802 (-)
Intergenic	MuAΔ61	99684-99688 (+)
UL48	MuAΔ71	99923-99927 (+)

	MuAΔ72	100650-100654 (-)
UL52	Tn5Δ17<br>MuAΔ57<br>MuAΔ67	105088-105096 (+)<br>105362-105366 (-)<br>105424-105428 (-)
UL53	MuAΔ68	108699-108703 (-)
Intergenic	MuAΔ60<br>MuAΔ77	108914-108918 (-)<br>111349-111353 (-)
LORF4A (HVT067)	Tn5Δ10	113752-113760 (-)
LORF5 (HVT069)	MuAΔ59<br>MuAΔ82	115165-115169 (+)<br>115602-115606 (-)
Intergenic	MuAΔ63<br><br>MuAΔ54<br><br>MuAΔ55	3898-3902 or<br>119785-119789 (ND)<br>1933-1937 or<br>121750-121754 (ND)<br>124295-124299 or<br>158297-158301 (ND)
vNR13 (HVT079/ HVT096)	MuAΔ46	124989-124993 or<br>157603-157607
icp4 (HVT080/ HVT095)	MuAΔ34<br><br>MuAΔ78<br><br>MuAΔ47<br><br>Tn5Δ19<br>MuAΔ83<br><br>Tn5Δ22<br>Tn5Δ4<br>Tn5Δ1<br>Tn5Δ3<br>Tn5Δ11<br>Tn5Δ8	128905-128909 or<br>153687-153691 (ND)<br>129979-129983 or<br>152612-152617 (ND)<br>130476-130480 or<br>152116-152120 (ND)<br>130947-130955 (+)<br>131154-131158 or<br>151438-151442 (ND)<br>132257-132365 (-)<br>149786-149794 (+)<br>151300-151308 (-)<br>153044-153052 (-)<br>154273-154281 (+)<br>154730-154738 (+)
Intergenic	MuAΔ56<br><br>MuAΔ25<br><br>MuAΔ51<br><br>MuAΔ44<br><br>MuAΔ50	133260-133264 or<br>149332-149336 (ND)<br>134298-134302 or<br>148294-148298 (ND)<br>134546-134550 or<br>148046-148050 (ND)<br>135789-135793 or<br>146803-146807 (ND)<br>135863-135867 or<br>146729-146733 (ND)
US3	MuAΔ70<br>MuAΔ45	141220-141224 (+)<br>141654-141658 (+)
US6	MuAΔ49	143203-143207 (-)
US8	Tn5Δ13<br>Tn5Δ21	145780-145788 (-)<br>145937-145945 (+)

	MuAΔ76	136053-135057 or 146539-146543 (ND)
Intergenic	Tn5Δ24	156371-156379 (-)
a-like sequences	Tn5Δ16<br>Tn5Δ20	Not determined
	MuAΔ65<br>MuAΔ84	BAC vector sequences

Supplemental Table S2: **Oligonucleotides used in this study.**

Oligonucleotide	Sequence (5' → 3')
<KAN2> FP1	ACCTACAACAAAGCTCTCATCAACC
KanMEFwd	CTCCTTCATTACAGAAACGGC
<KAN2> RP1	GCAATGTAACATCAGAGATTTTGAG
KanMERev	ACAATCGATAGATTGTCGCAC
HVT27Fwd	CGAAAGCGGATAACCAGAAC
HVTFrag5Rev	AAATCGCCATTTTACGCATC
KanRMfeIFwd	TGAGTTAACGTCGGTTGATGAGAGCTTTGTTGTAGGTG
GFPseq	CGTCGCCGTCCAGCTCGACCAG
ECGFPH Fwd	GTTCCTAAGCTTTTGCTGGCCTTTTGCTCACATGT

Supplemental Figure S1: **Primary chicken embryo fibroblasts (CEFs) were used as a negative control**

Primary chicken embryo fibroblasts (CEFs) were monitored as a negative control to assess the replication capacity of transposition mutants in cell culture. Day 7 of passage 1 a: brightfield ×100; and b: fluorescent microscopy ×100

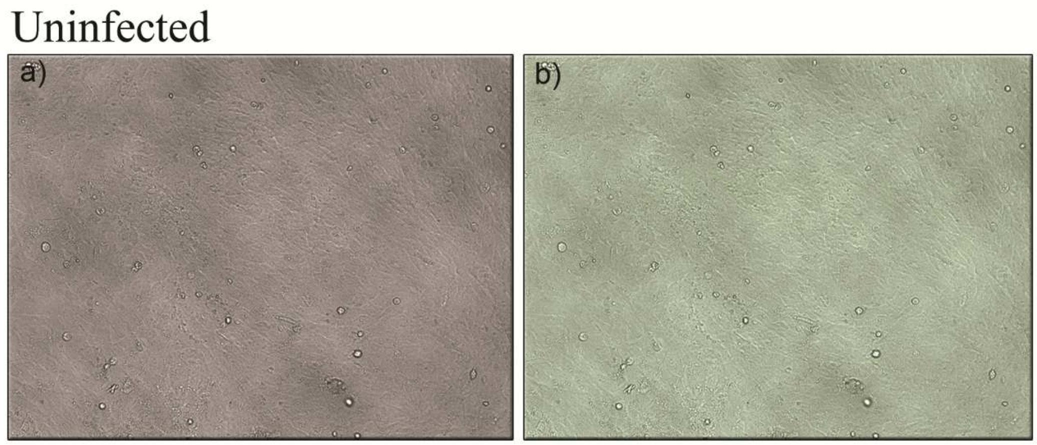

Supplement: Additional file 1: — Supplemental Table S1. Details of transposon gene interruptions into the MeHV-1 infectious clone pMeHV1-C18. Supplemental Table S2: Oligonucleotides used in this study. Supplemental Figure S1: Primary chicken embryo fibroblasts (CEFs) were used as a negative control. (PDF 121 kb) [file 12985_2015_362_MOESM1_ESM.pdf]
